# Contrasting patterns of nickel distribution in the hyperaccumulators *Phyllanthus balgooyi* and *Phyllanthus rufuschaneyi* from Malaysian Borneo

**DOI:** 10.1093/mtomcs/mfac020

**Published:** 2022-03-26

**Authors:** Antony van der Ent, Jolanta Mesjasz-Przybyłowicz, Wojciech J Przybyłowicz, Alban D Barnabas, Martin D de Jonge, Hugh H Harris

**Affiliations:** Centre for Mined Land Rehabilitation, Sustainable Minerals Institute, The University of Queensland, St Lucia 4072, Australia; Department of Botany and Zoology, Stellenbosch University, Matieland 7602, South Africa; Department of Botany and Zoology, Stellenbosch University, Matieland 7602, South Africa; Faculty of Physics & Applied Computer Science, AGH University of Science and Technology, 30-059 Kraków, Poland; Materials Research Department, iThemba LABS, National Research Foundation, Somerset West 7129, South Africa; Australian Synchrotron, ANSTO, Clayton 3168, Australia; Department of Chemistry, The University of Adelaide, Adelaide 5005, Australia

**Keywords:** elemental mapping, hyperaccumulator, phloem, micro-PIXE, nuclear microprobe, synchrotron X-ray fluorescence microscopy

## Abstract

Globally, the majority of Ni hyperaccumulator plants occur on ultramafic soils in tropical regions, and the genus *Phyllanthus*, from the Phyllanthaceae family, is globally the most represented taxonomical group. Two species from Sabah (Malaysia) are remarkable because *Phyllanthus balgooyi* can attain >16 wt% of Ni in its phloem exudate, while *Phyllanthus rufuschaneyi* reaches foliar concentrations of up to 3.5 wt% Ni, which are amongst the most extreme concentrations of Ni in any plant tissue. Synchrotron X-ray fluorescence microscopy, nuclear microbe (micro-PIXE+BS) and (cryo) scanning electron microscopy with energy dispersive spectroscopy were used to spatially resolve the elemental distribution in the plant organs of *P. balgooyi* and *P. rufuschaneyi*. The results show that *P. balgooyi* has extraordinary enrichment of Ni in the (secondary) veins of the leaves, whereas in contrast, in *P. rufuschaneyi* Ni occurs in interveinal areas. In the roots and stems, Ni is localized mainly in the cortex and phloem but is much lower in the xylem. The findings of this study show that, even within the same genus, the distribution of nickel and other elements, and inferred processes involved with metal hyperaccumulation, can differ substantially between species.

## Graphical Abstract

**Graphical Abstract:**
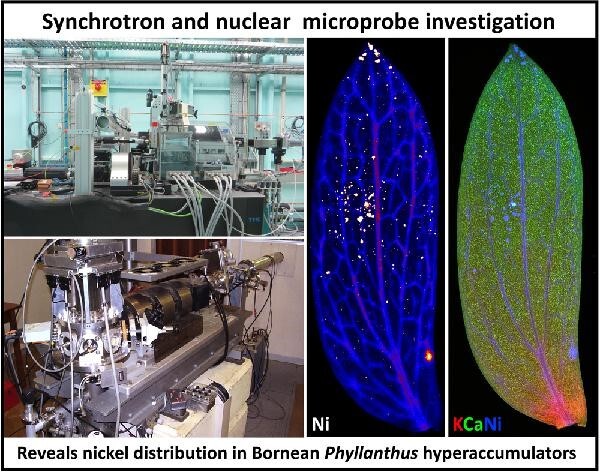
Synchrotron and nuclear microbe techniques were used to unravel the distribution of nickel and other elements at the organ and tissue level of the hyperaccumulators *Phyllanthus rufuschaneyi* and *Phyllanthus balgooyi* from Sabah, Malaysia.

## Introduction

Even though nickel (Ni) is essential to plants at very low concentrations (0.05–10 μg g^–1^), the range between deficient and toxic levels is rather wide.^1^ Toxicity of Ni causes oxidative and genotoxic stresses visible as foliar chlorosis that ultimately depresses plant growth.^2,3^ Therefore, plants effectively regulate Ni homeostasis by controlling root uptake and translocation to the shoots. Most plants growing on ultramafic soils (naturally enriched in Ni) exclude Ni from uptake, whilst a very small number are hyperaccumulators capable of accumulating Ni to extremely high concentrations in plant shoots.^4^^--^^6^, The highest Ni concentrations in plants found thus far include 7.6 wt% Ni in leaves of the South African *Berkheya coddii* Roessler^7^ and 25 wt% in the latex of *Pycnandra acuminata* (Pierre ex Baill.) Swenson and Munzinger from New Caledonia.^4^ The degree of bioconcentration is remarkable in these plants, e.g. many hyperaccumulators accumulate >2 wt% foliar Ni from soils with just 0.1 wt% total Ni.^8^ The highly enhanced translocation in the shoot results from mechanisms for translocating Ni towards the shoot from the root.^9^ The fundamental biomolecular processes that regulate Ni in plants are poorly understood, although it is assumed that Ni hyperaccumulation evolved from the analogous mechanisms that regulate zinc (Zn), manganese (Mn), and/or iron (Fe) homeostasis with a strong modification of three essential steps: (i) uptake of Ni by roots; (ii) effective translocation of Ni from root to the shoot, including radial transport to and from vascular tissues; and (iii) detoxification and sequestration of Ni in foliar cells.^10^ Phloem redistribution has been shown to be involved in Ni fluxes and re-distribution between old and young leaves.^11^

Information on the fundamental physiological mechanisms of Ni accumulation is useful for efforts to select and breed better ‘metal crops’ for application in phyto/agromining. This is an emerging approach that utilizes hyperaccumulator plants to obtain Ni from ultramafic soils.^12–14^ There is a strong incentive for Ni agromining to mitigate some of the negative consequences of conventional strip-mining operations in Indonesia and New Caledonia. In excess of 500 Ni hyperaccumulator species (>0.1 wt% in shoots) are now known, but only ∼50 hypernickelophores (plant taxa with >1 wt% in their shoots) have been discovered, whilst these have the greatest potential for phyto/agromining.^15–17^ The majority of known hypernickelophores originate from Cuba,^18^ New Caledonia,^19^ and Southeast Asia.^20^ Among the most promising of these species are several taxa in the genus *Phyllanthus* (Phyllanthaceae) that often grow fast and have preferable growth characteristics for cultivation, including ease of mass propagation and herbivore resistance.^13,14^ However, to date, very little scientific inquiry has been devoted to tropical Ni hyperaccumulator plant species from Malaysia and Indonesia.^20^

Nickel hyperaccumulation is a particularly distinctive attribute of the Malpighiales and is frequent in the families Dichapetalaceae, Phyllanthaceae, Salicaceae, and Violaceae.^21^ By far, the Phyllanthaceae has the greatest number of hyperaccumulator plant taxa that are known from the *Actephila, Antidesma, Breynia, Cleistanthus, Glochidion*, and *Phyllanthus* genera. The genus *Phyllanthus* has >800 species and is especially diversified in New Caledonia (113 species), Cuba (50 species), and Southeast Asia (120 species).^22^ In New Caledonia, 14 *Phyllanthus* species are Ni hyperaccumulators,^23,24^ whilst in Cuba, 19 *Phyllanthus* species are Ni hyperaccumulators.^24^*Phyllanthus* species are known to attain amongst the highest Ni concentrations of all hyperaccumulating plants, with 4.2 wt% in *P. favieri* M.Schmid (synonym *P. serpentinus)* from New Caledonia^19^ and 6 wt% in *P.* × *pallidus* from Cuba.^18^ Additional genera within the Phyllanthaceae continue to yield new Ni hyperaccumulator records, e.g. *Antidesma montis-silam* Airy Shaw,^25^ as well as novel taxa that are hyperaccumulating, including *Actephila alanbakeri* Welzen and Ent.^26^ Sabah (Malaysia) in the island of Borneo is a major centre for diversity for hyperaccumulator plants with eight species of *Phyllanthus*, including: *P. balgooyi* Petra Hoffm. & A.J.M. Baker and *Phyllanthus rufuschaneyi* Welzen, R.W.Bouman and Ent.^27^ In earlier studies,^8,27,28^ the latter taxon was initially identified as *Phyllanthus securinegoides* Merr. because it resembled this taxon from the Mindanao in the Philippines.^29^ However, it was more recently described as the novel taxon *P. rufuschaneyi* (Phyllanthaceae).^30^ Apart from the aforementioned *P. balgooyi* and *P. securinegoides*, a third hyperaccumulating *Phyllanthus* species also occurs in the Philippines; *P. erythrotrichus* C.B.Rob., with up to 1.1 wt% Ni in the leaves.^31^

*Phyllanthus balgooyi* is capable of accumulating up to 16.9 wt% Ni in the phloem sap and up to 0.86 wt% in the leaves, while *P. rufuschaneyi* can accumulate up to 3.5 wt% Ni in leaves and 1.8 wt% in the phloem tissue.^27,32^ In leaves, Ni^2+^ is mainly complexed by carboxylic acids such as citrate.^28^,^33–35^ Earlier, we have performed synchrotron X-ray absorption spectroscopy (XAS) on *P. balgooyi* and *P. rufuschaneyi*, which showed that Ni is complexed with carboxylic acids (mainly citrate) throughout the plants, from roots to stems and leaves, as well as in transport liquid (xylem and phloem).^28^ Previous investigations using micro-particle-induced X-ray emission (PIXE) showed that the phloem of the stem and petiole of *P. balgooyi* acts as a ‘sink’ with Ni reaching up to 9.4 wt% and 10.3 wt%, respectively. In the leaves, Ni was highly enriched in the vascular bundles (up to 8.9 wt%), while in the upper epidermis it was up to 1.3 wt%. Minor Ni enrichment was also noted in the lower epidermis.^36^ In *P. rufuschaneyi*, Ni is also strongly enriched in the phloem, with up to 5.6 wt% in the phloem bundles of the root, whereas in the leaves, the upper epidermis is notably richer in Ni than in *P. balgooyi* (up to 4 wt% Ni on average).^28^

The current investigations aim to build on the published results^28,36^ and to take advantage of X-ray fluorescence microscopy (XFM) for its high resolution (∼1 μm here) and the capability to scan very large samples (up to 100 × 150 mm) generating megapixel maps.^37,38^ We have again used PIXE on cross-sections of roots, stems, and leaves to exploit its sensitivity for light element (Al, Cl, Si, S, and P) analysis and accurate quantification with proton backscattering spectrometry (BS). This was complemented by examination of frozen-hydrated tissue cross-sections (cryo) scanning electron microscopy with energy dispersive spectroscopy (SEM-EDS). This study directed, therefore, to unravel the distribution of Ni and other macro and micro elements (Ca, K, Mn, and Zn) at the whole organ level (i.e. entire leaves, inflorescences).

## Materials and methods

### Occurrence of *P. balgooyi* and *P. rufuschaneyi* in Sabah

**Figure 1.**
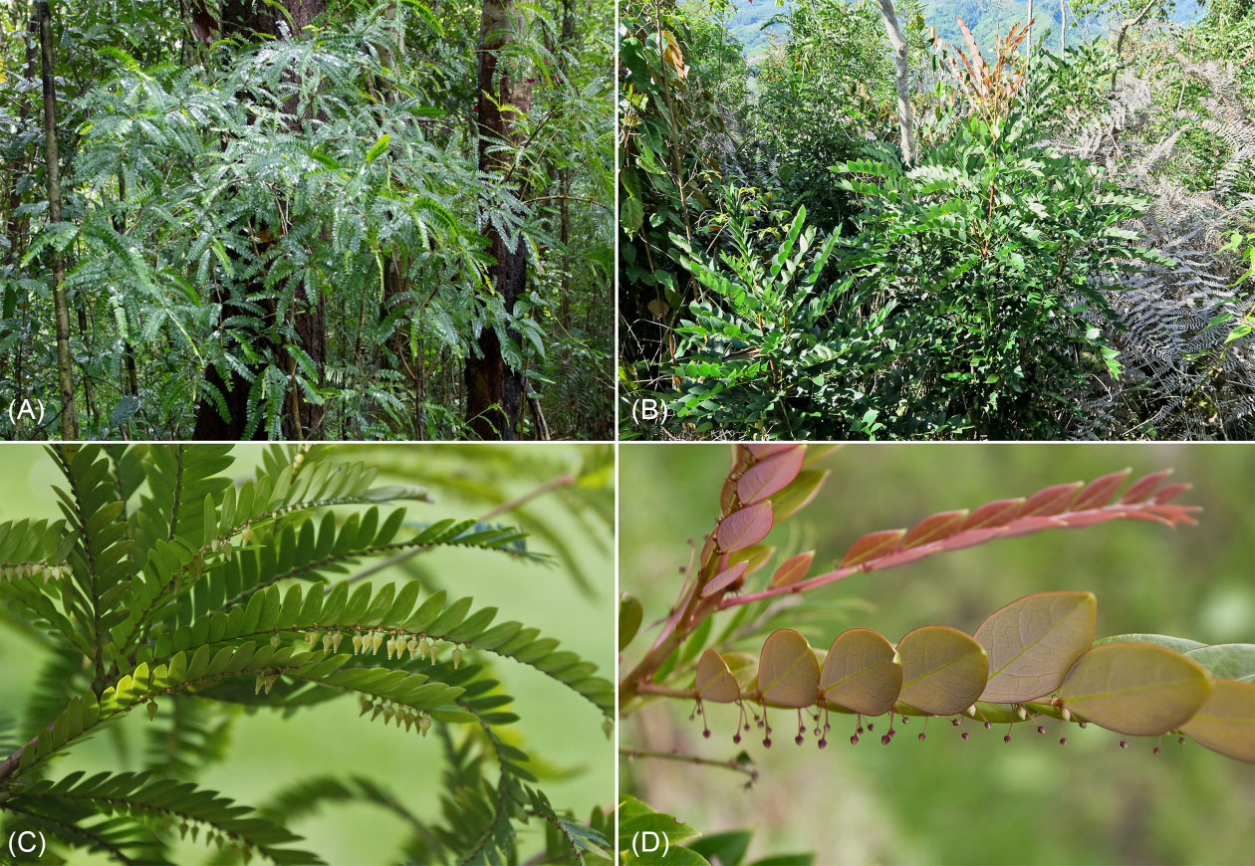
Plants growing in the native habitats in Sabah (Malaysia): (A) *P. balgooyi* is an understorey tree; (B) *P. rufuschaneyi* is a shrub from secondary vegetation; (C) close-up of *P. balgooyi* individual flowers borne in pairs in leaf axils; and (D) close-up of *P. rufuschaneyi* flowers in fascicles of multiple flowers.

*Phyllanthus balgooyi* (Phyllanthaceae) was originally discovered to be a Ni hyperaccumulator in Palawan (Philippines). It grows on mountain ridges as a small shrub up to ∼1.5 m high.^29^ It also occurs in Sabah, where it can grow up to 8 m tall with a bole up to 25 cm in diameter (Fig. 1). *Phyllanthus balgooyi* has phyllanthoid branches with closely distichous leaves (20–70 per branchlet) measuring 0.7–1.5 × 0.3–0.6 cm.^39^*Phyllanthus rufuschaneyi* (Phyllanthaceae) was discovered as a Ni hyperaccumulator in Sabah where it accumulates up to 3.5 wt% Ni in leaves.^32^ It is a multi-stemmed shrub or treelet up to 9–10 m tall with phyllanthoid branches with spaced leaves (10–15 per branchlet) measuring 1.0–2.5 × 1.5–3 cm each (Fig. 1). Staminate and pistillate flowers in both species emerge throughout the year, are numerous, small (1.5–2 × 2–3 mm) and borne on the branches in the axils of the leaves. *Phyllanthus balgooyi* and *P. rufuschaneyi* differ in their ecological niches, whereas *P. balgooyi* occurs in the primary (undisturbed) rainforest, *P. rufuschaneyi* occurs in disturbed secondary scrub, particularly after fire. *Phyllanthus balgooyi* ostensibly has a slow growth rate, whereas *P. rufuschaneyi* is a fast-growing pioneer of open areas. The chemistry of the rhizosphere soil associated with *P. balgooyi* and *P. rufuschaneyi* has been outlined before in detail^8,26,32^ and is distinguished by high phyto-available Ni content and a near-neutral pH.

### Collection of samples, bulk elemental analysis, and preparation for XFM and micro-PIXE

Plant material samples (flower, stem, twig, leaf, phloem tissue, fruit, and seed) were harvested in the natural habitats in Sabah, Malaysia. The leaves, fruits, and flowers were simply excised with scissors. Seeds were extracted from the fruit. The phloem tissue was stripped from the bark using a razor blade. The stem (lignified and brown, 2–5 mm diameter) and twigs (green and soft, 2–3 mm diameter) were cut from the apical portion of the branches. These samples were dried at 70°C in a drying oven and subsequently ground and digested using 4 ml HNO_3_ (70%) in a microwave oven (Milestone Start D) for a 45-min programme and diluted to 30 ml and analysed with inductively coupled plasma-atomic emission spectroscopy (ICP-AES) (Varian Vista Pro II), as described previously.^40^ Tissue samples of *P. balgooyi* (Phyllanthaceae) and *P. rufuschaneyi* (Phyllanthaceae) were collected near Serinsim, on the northern edge of Kinabalu Park in Sabah, Malaysia. Individual tissue samples for synchrotron XFM and nuclear microprobe (micro-PIXE) analysis were fast frozen by the metal mirror technique, transported in a liquid nitrogen vapour cryogenic Dewar, and freeze-dried in a Leica EM CFD Cryosorption Freeze Dryer (Leica Microsystems AG, Austria), following an earlier protocol.^40^ Properly executed freeze-drying (lyophilization) does not lead to structural changes or elemental distribution, even at the cellular scale.^37^,^41–43^

### Light microscopy and SEM and cryogenic SEM-EDS analysis

Plant tissue specimens of mature leaves were first fixed in 3% glutaraldehyde and then post-fixed in 2% osmium tetraoxide (OsO_4_). Following that, the specimens were dehydrated in an ethanol series and embedded in Spurr's resin. Finally, the specimens were sectioned and stained with Azur II/methylene blue for imaging with a microscope, following the earlier described protocol.^44^ Freeze-dried leaf specimens were carbon coated, mounted on stubs, and imaged with scanning electron microscopy (SEM) with X-ray microanalysis (SEM-EDS) on a JEOL JSM-6610 instrument (with a 50 mm^2^ Oxford Instruments SDD detector), as described previously.^40^ CryoSEM-EDS was undertaken using a JEOL JSM-7100F instrument on frozen-hydrated specimens, as described previously.^45^ The reported concentration values are semi-quantitative.^46^

### Synchrotron XFM and nuclear microprobe PIXE analysis

The XFM beamline of the Australian Synchrotron has an in-vacuum undulator to produce an X-ray beam with an energy of 4.1–20 keV that can be focused to 1000 nm.^47^ The incident energy used was 15.8 keV. The P06 beamline of PETRA III [Deutsches Elektronen-Synchrotron (DESY)] is also equipped with Si(111) monochromator and K/B mirrors^48^ producing an X-ray beam with an energy of 5–23 keV that can be focused to 300 nm. The incident energy used was 11 keV. The XFM and P06 beamlines are both equipped with a Maia detector.^49,50^ The beamline experimental conditions and processes for data acquisition have been described in detail previously in other studies by our group.^28,40,45,51^

The nuclear microprobe of iThemba LABS (South Africa) produces a proton beam of 3 MeV energy from a 6 MV single-ended Van de Graaff accelerator that is focused on a 3 × 3 μm^2^ spot.^52,53^ PIXE and proton BS were used simultaneously, and the PIXE data were collected using an Si(Li) detector (30 mm^2^ with a 125 μm Be layer absorber), whilst the BS data were collected with an annular Si surface barrier detector (100 μm thick). The experimental parameters and procedures for PIXE analysis of plant specimens have been detailed in earlier publications by our group.^28,36,44,54^ The micro-PIXE and XFM data were processed using the GeoPIXE software.^55–59^

## Results

### Bulk chemistry of *P. balgooyi* and *P. rufuschaneyi* tissues

Bulk elemental analysis using ICP-AES of the foliar samples of *P. balgooyi* and *P. rufuschaneyi* confirmed the hyperaccumulation status with up to 1 wt% and 2.5 wt% foliar Ni, respectively (Table 1). Calcium concentrations are also high, particularly in *P. rufuschaneyi*, reaching up to 1.1 wt% in the leaves and up to 3.6 wt% in the phloem tissue. The amount of K in the leaves, twigs, and phloem of both species is rather high (up to 1.1 wt% in *P. balgooyi* leaves, and 1.4 wt% in twigs of *P. rufuschaneyi*), considering that these plants grow on severely K-deficient ultramafic soils. The faster growth rate of *P. rufuschaneyi* compared to *P. balgooyi* might explain some of the differences in macro-element concentrations. The flowers, fruits, and seeds of *P. rufuschaneyi* have high Ni concentrations (2900–4000 μg g^–1^). Other elements are unremarkable with Al, Co, Fe, Mn, and Zn in the typically expected ranges (compare with values cited in van der Ent et al. 2015).

**Table 1.** Bulk elemental concentrations in plant tissues (flowers, leaves, twigs, and phloem) in *P. balgooyi* and *P. rufuschaneyi.* Macro and trace elements (Al, Ca, Co, Fe, K, Mg, Mn, Ni, P, S, and Zn). Values as ranges and means in μg g^–1^ dry weight

Species	*n*	Al	Ca	Co	Fe	K	Mg	Mn	Ni	P	S	Zn
*Phyllanthus*	**Flowers**											
*balgooyi*	1	24.4	1479	21	18	6630	3826	12	736	1136	1695	23
	**Stem**											
	1	9.4	1691	31	21	246	433	259	2978	83	379	45
	**Leaf**											
	10	56	4932	27.6	117.6	6152	6904	95	3315	1545	1658	49
		10–121	3018–7303	4.4–60	23–231	2767–10 534	3512–10 946	49–290	517–9889	281–2763	725–2299	29–72
	**Phloem tissue**											
	3	16	3685	682	15	2841	988	207	72212	240	2028	1146
		12–17	2916–4408	193–1170	9.1–20.5	2701–3017	709–1164	162–283	62 183–79 342	234–244	1782–2154	720–1933
	**Twig**											
	2	5.7	381	9.8	11.3	1055	304	12.0	1501.0	77.9	251.4	21.8
		1.3–10.1	130–633	8.3–11	4.0–19	294–1816	109–500	4.9–19	452–2550	16–139	181–322	8.5–35
*Phyllanthus*							**Flowers**					
*rufuschaneyi*	1	20.4	3327	16	24	4604	2813	34	2905	1071	904	20
	**Fruit**											
	2	16.9	3612	10.5	27	5685	1635	60	3651	1188	1206	21
		9.3–24	3063–4161	7.7–13	23–30	5448–5922	1468–1802	38–83	3301–4001	1131–1245	1021–1391	21–21
	**Seeds**											
	1	26.2	4478	22.6	30.5	6011	2737	64	1421	3333	1940	26.3
	**Stem**											
	1	7.3	5733	15	11	1318	377	47	3478	188	445	42
	**Leaf**											
	12	26	5585	46	62	7379	3744	147	11 902	697	2241	38
		11–52	2190–10 920	22–89	22–136	4158–10 240	2033–6896	72–281	1105–25 057	473–939	1199–3612	16–84
	**Phloem tissue**											
	1	29.4	36 410	21.0	59.6	6399	1355	62	9337	339	1100	190.7
	**Twig**											
	2	32.7	4375	18	22	10 101	1142	90	6443	852	948	63
		7.8–71	1176–6892	15–20	14–38	4915–13 678	836–1750	60–151	878–12 309	252–1727	323–1716	42–87

### Anatomical features of the roots, stems, and leaves

**Figure 2.**
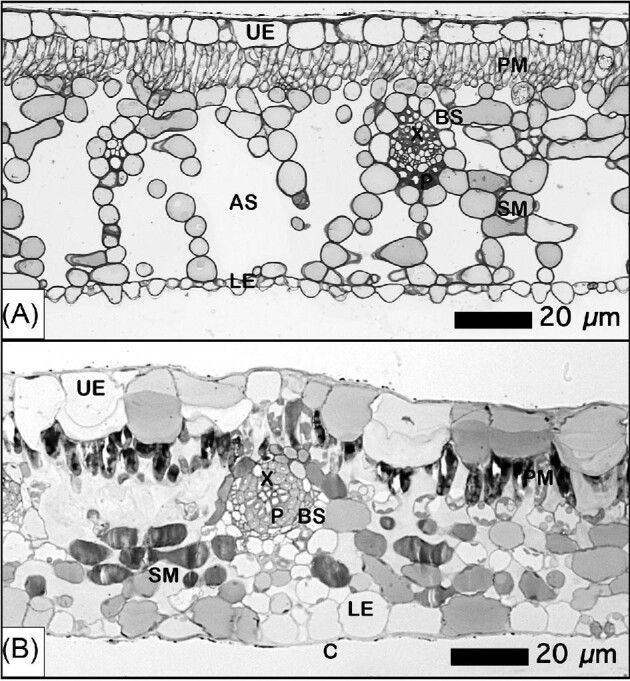
Azur II and methylene blue stained (in greyscale for better contrast and clarity) leaf blade transverse section of *P. balgooyi* (A) and *P. rufuschaneyi* (B). Abbreviations: *UE* upper epidermis, *LE* lower epidermis, *C* cuticle, *PM* palisade mesophyll, *SM* spongy mesophyll, *AS* air space, *BS* bundle sheath, *X* xylem, and *P* phloem.

**Figure 3.**
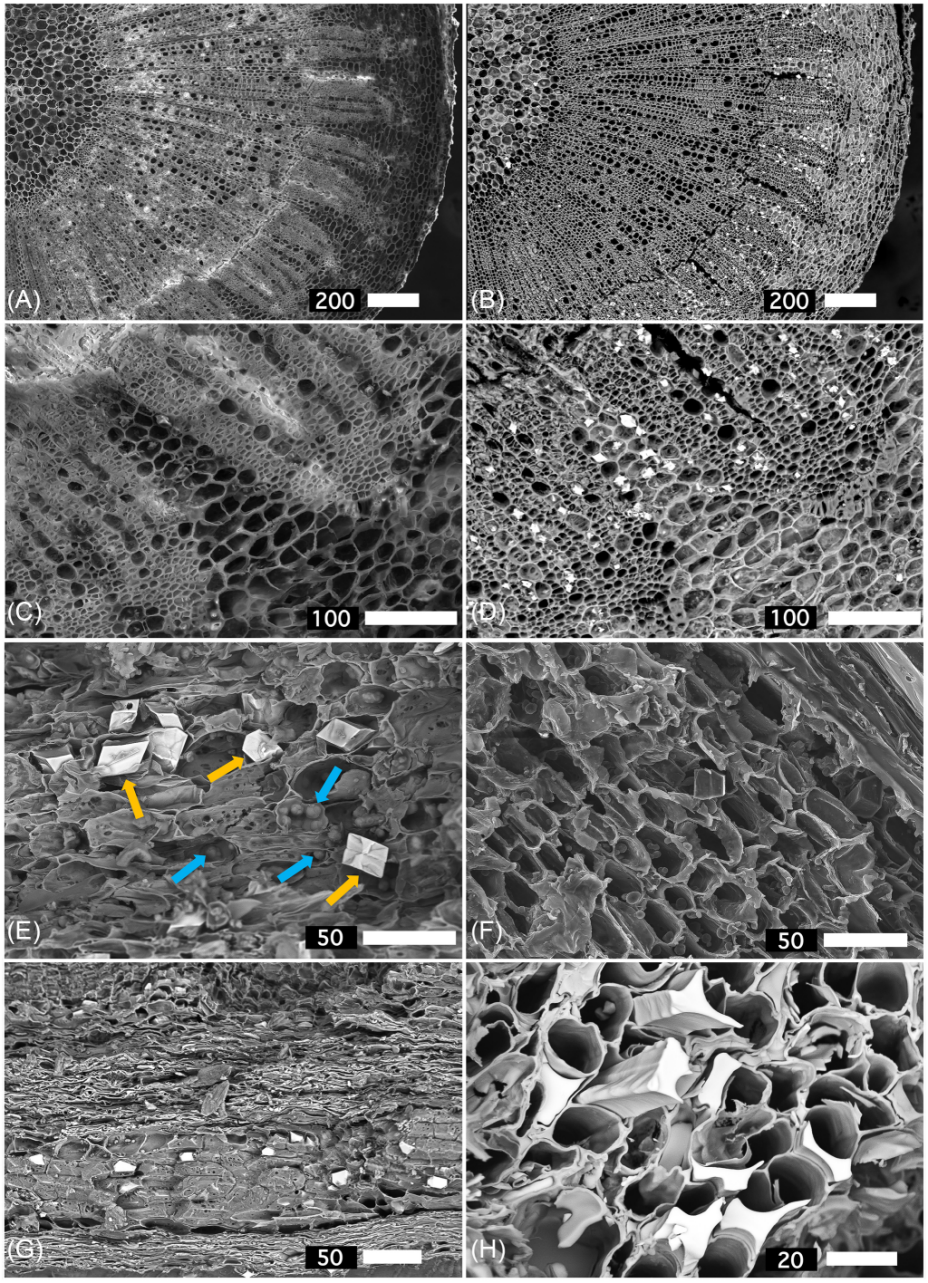
Scanning electron microscopy (SEM) images of *P. balgooyi:* (A) secondary electron (SE) image of root cross-section; (B) back-scattered electron (BSE) image of the same root cross-section; (C) close-up of *A*; (D) close-up of *B* showing abundant Ca-oxalate crystals; (E) SE image of phloem tissue showing Ca-oxalate crystals (orange arrows) and Ni-rich globules (blue arrows); (F) detail of the same phloem tissue showing sieve elements; and (G) BSE image of wood; and (H) BSE image close-up showing Ni-rich precipitates.

*Phyllanthus balgooyi* has large regularly sized square adaxial (upper) epidermal cells, whereas the epidermal cells on the abaxial (lower) side of the leaf are small and irregularly shaped (Fig. 2). In *P. rufuschaneyi*, the epidermal cells are even larger, ovoid, and of similar size on the adaxial and abaxial sides of the leaf (Fig. 2). Whereas *P. balgooyi* has a very dense palisade mesophyll, in *P. rufuschaneyi* the cells are more scattered. In contrast to *P. rufuschaneyi*, the spongy mesophyll in *P. balgooyi* is extremely open with large air spaces. The vascular bundles of the mid-vein and lateral veins in the mesophyll consist of phloem and xylem vessels enclosed by bundle sheath cells. SEM was undertaken on various dehydrated *P. balgooyi* tissues (Fig. 3). The secondary electron (SE) image of the phloem tissue revealed abundant Ca-oxalate crystals (panel E, orange arrows) and Ni-rich globules (blue arrows). The latter are precipitated Ni-citrate deposits. Panel (F) shows a detail of the same phloem tissue showing sieve elements. Ni-rich precipitates are also visible in the back-scattered electron (BSE) image of the wood (panel G), with a further close-up (panel H). In the SEM images of a root cross-section, calcium-oxalate crystals are abundant in medullary rays extending from the xylem (panels A–D).

### Scanning electron microscopy for subcellular nickel localization

**Figure 4.**
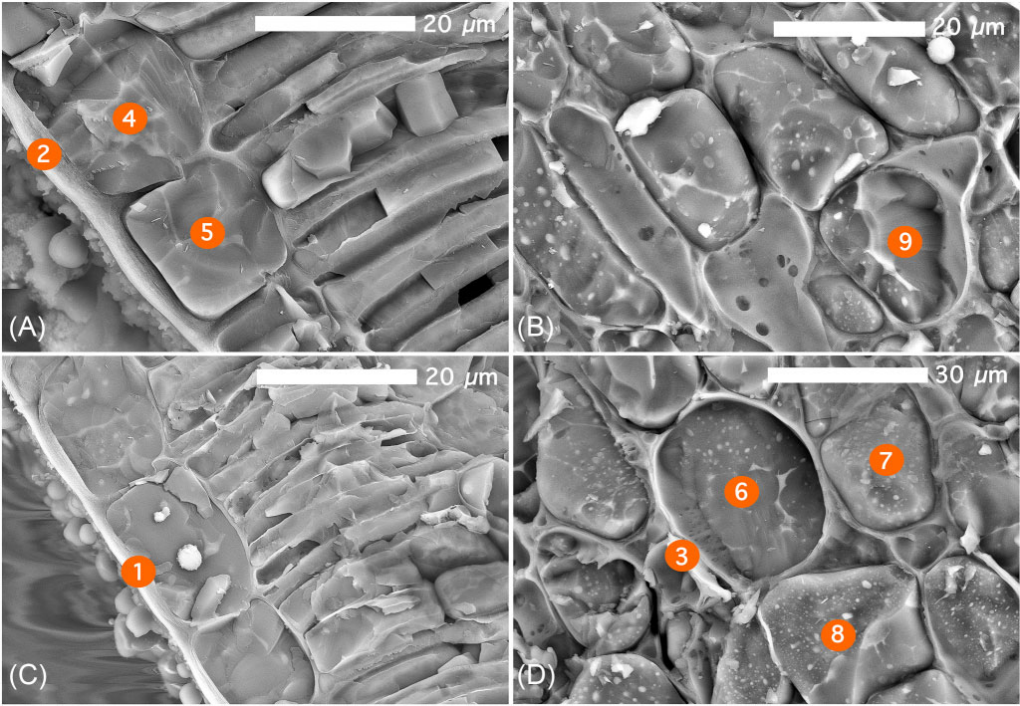
Cryogenic scanning electron microscopy (cryoSEM) images of *P. balgooyi* showing: (A) upper epidermis with cuticle, epidermal cells, and mesophyll visible; (B) mesophyll cells, note very wide apoplastic space; (C) further upper epidermal cells; and (D) mesophyll cells. Individual numbers 1–9 marked in orange circles correspond to energy-dispersive spectroscopy (EDS)-point analysis in Table 2.

Frozen-hydrated *P. balgooyi* foliar fragments were cryofractured and point energy-disperse spectroscopy (EDS) analysis in an electron microscope was undertaken to determine Ni localisation at the (sub)cellular scale (Fig. 4). Panels (A and C) show the lower epidermal region of the leaf, whereas panels (B and D) show a portion of the underlying mesophyll. At 20 kV accelerator voltage, the maximum penetration depth of the e^–^ beam is ∼20 μm and the horizontal resolution <1 μm. In theory, these permits obtaining differential measurements of the cell wall/apoplast and of the vacuole. High O over C mass % is indicative of the hydration state, e.g. the amount of water, and hence vacuolar contents. Oxygen content in the vacuoles ranges from 74.6 to 94.1 wt%, predictably much higher than in the cell walls and apoplasts, where it is between 62.5 and 68.6 wt%. The Ni concentration in the vacuoles is between 0.6 and 2.1 wt%, significantly higher than in the cell walls and apoplasts, where it does not exceed 0.5 wt%. Calcium and K concentrations are higher in the cell wall than in the vacuole areas (Table 2).

**Table 2.** EDS concentration values obtained via cryo SEM of fractured frozen-hydrated *P. balgooyi* leaf fragments. Values are reported as mass % (total of atom count is 100% excluding Pt) with errors

	Element	C	O	K	Ca	Ni
	keV	0.277	0.525	3.312	3.69	7.471
*Phyllanthus balgooyi*	Point #	Mass%	Mass%	Mass%	Mass%	Mass%
Cell wall & apoplast	1	32.9 (± 0.04)	62.5 (± 0.07)	0.7 (± 0.04)	0.3 (± 0.04)	0.3 (± 0.2)
	2	24.2 (± 0.07)	67 (± 0.09)	1.1 (± 0.06)	0.4 (± 0.06)	0.3 (± 0.2)
	3	29.8 (± 0.03)	68.6 (± 0.06)	0.4 (± 0.03)	0.5 (± 0.04)	0.5 (± 0.2)
Vacuole	4	21.6 (± 0.04)	77.3 (± 0.06)	0.3 (± 0.05)	0.05 (± 0.05)	0.4 (± 0.2)
	5	13 (± 0.06)	84.1 (± 0.06)	0.7 (± 0.06)	0.1 (± 0.06)	0.6 (± 0.3)
	6	4.2 (± 0.10)	94.1 (± 0.07)	0.3 (± 0.10)	0.3 (± 0.1)	1 (± 0.5)
	7	5.2 (± 0.08)	93.3 (0.06)	0.3 (± 0.08)	0.05 (± 0.09)	1.2 (± 0.4)
	8	3.3 (± 0.01)	94.1 (0.07)	0.3 (± 0.1)	0.02 (± 0.1)	2.1 (± 0.4)
	9	23.9 (± 0.04)	74.6 (0.06)	0.30 (± 0.04)	0.4 (± 0.04)	0.6 (± 0.2)

### Elemental distribution in various tissues revealed by XFM and PIXE

The result of this study complies with earlier investigations^28,36^ and reveals that *P. balgooyi* Ni has extreme levels of Ni accumulation in the vascular tracts and phloem bundles. When the trunk is damaged, *P. balgooyi* produces copious amounts of a dark green liquid that contains Ni at up to 16.9 wt%.^32^*Phyllanthus rufuschaneyi* also has Ni-rich phloem and vascular bundles but does not produce appreciable amounts of phloem sap. The extremely high concentrations of Ni in the phloem are observed throughout *P. balgooyi*, from the trunk to the phloem cells in the leaves.

**Figure 5.**
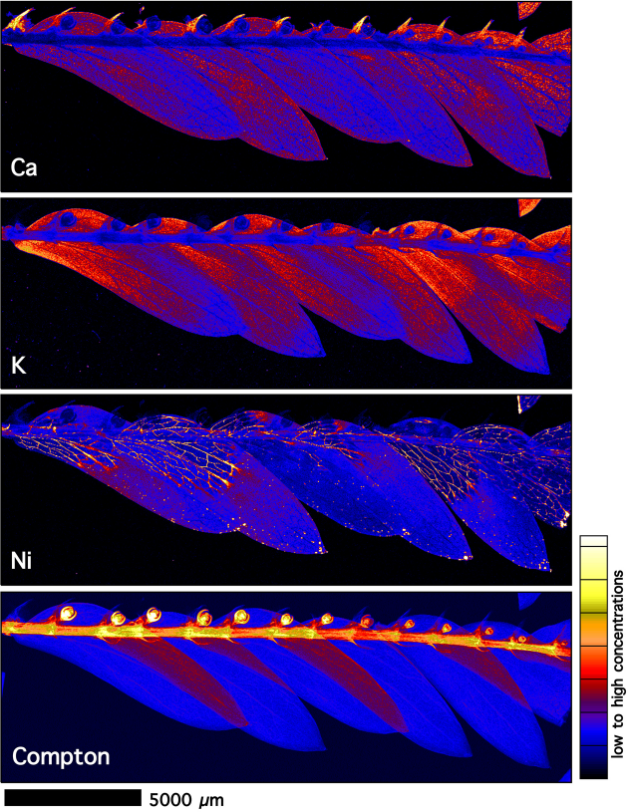
Individual elemental micro-X-ray florescence (μXRF) maps (Ca, K, Ni, and Compton Scatter) of freeze-dried *P. balgooyi* branchlet with inflorescences. The elemental image was acquired in a 2-μm step size with a 2.6 ms dwell per pixel. Acquired at the X-ray fluorescence microscopy (XFM) beamline of the Australian Synchrotron (ANSTO).

**Figure 6.**
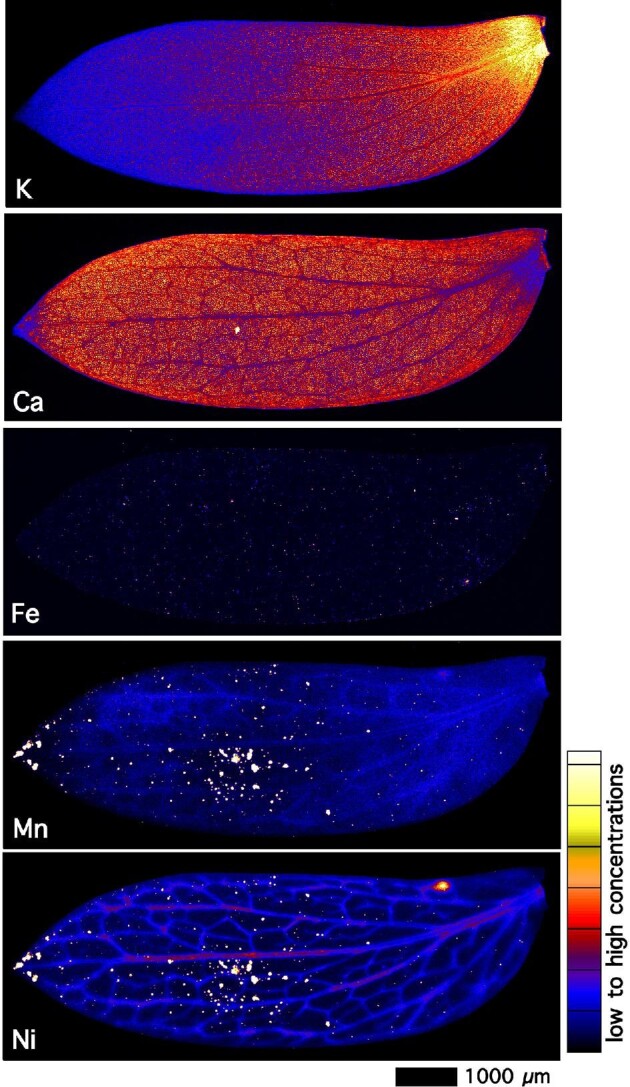
Elemental micro-X-ray florescence (μXRF) maps of whole freeze-dried *P. balgooyi* leaf showing K, Ca, Mn, and Ni distribution. The elemental image measuring 9.8 × 3.7 mm in area was acquired in an 8-μm step size with a 10 ms dwell per pixel. Acquired at the P06 beamline of the German Synchrotron (DESY).

Elemental maps of a freeze-dried *P. balgooyi* branchlet with inflorescences (Fig. 5) show major enrichment of Ni in the primary and secondary veins in the phyllanthoid branch into the leaflets, whereas Ca is present across the leaflets and especially in the inflorescences. In the whole leaves of *P. balgooyi* (Fig. 6), Ni is distributed throughout, but with some enrichment in the main vascular bundles. Across the leaf, small hotspots occur, particularly towards the leaf tip, which are strongly enriched in Ni and Mn. These hotspots are not likely to be soil particles because they are not enriched in soil-rich elements, such as Fe or Cr, and may be deposits originating from guttation fluid expelled *via* water pores (hydathodes).

**Figure 7.**
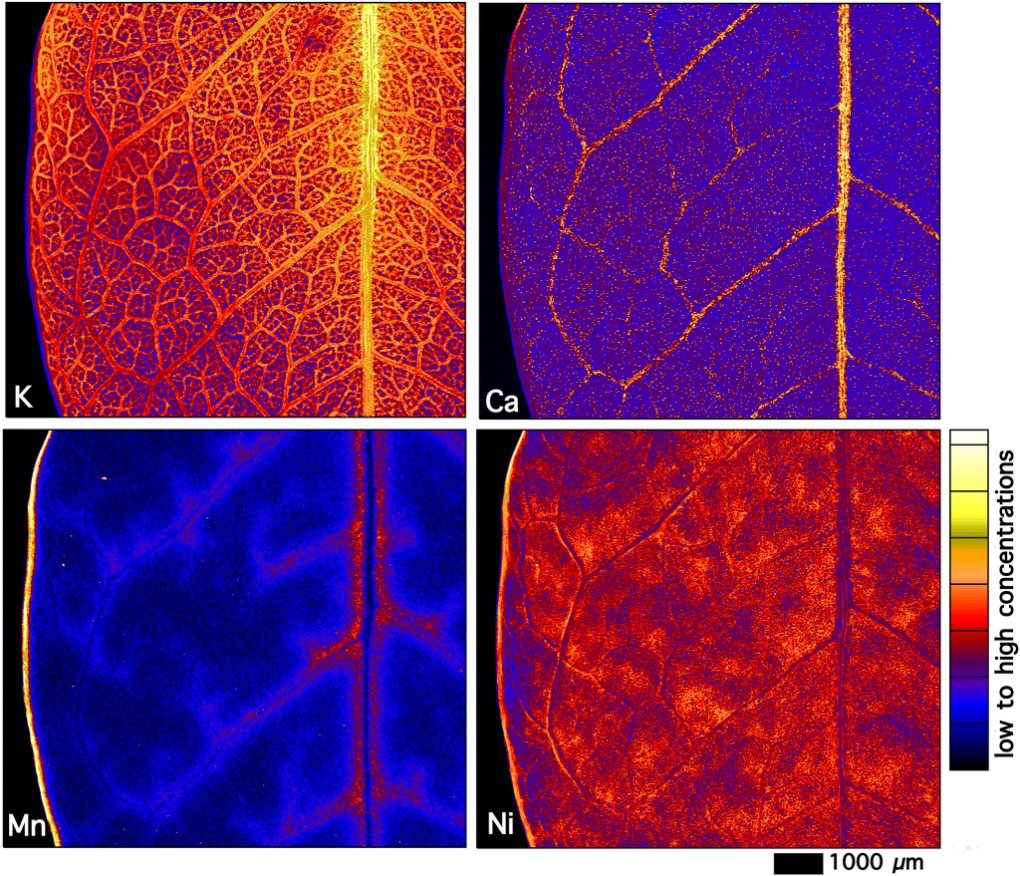
Elemental micro-X-ray florescence (μXRF) maps of the central portion of freeze-dried *P. rufuschaneyi* leaf showing K, Ca, Mn, and Ni distribution. The elemental image measuring 9.1 × 8.2 mm in area was acquired in a 15-μm step size with a 10 ms dwell per pixel. Acquired at the P06 beamline of the German Synchrotron (DESY).

In the whole leaves of *P. balgooyi* (Fig. 6), Ca is diminished in the vascular bundles and in the interveinal areas of the leaf (lamina). The distribution of Ca in *P. rufuschaneyi* (Fig. 7) is very different. There is an enrichment in the vascular bundles and in many very small (<5 μm) hotspots occurring evenly over the leaf. These Ca hotspots appear to coincide with abundant globular papillary type trichomes (∼5 μm in diameter). Nickel is distributed throughout the leaf but depleted in the main vascular bundles (Fig. 7). The concentrations of Co are very low (<100 μg g^–1^), and apart from a few minuscule hotspots around the leaf margin, no distribution patterns can be observed (map not shown).

**Figure 8.**
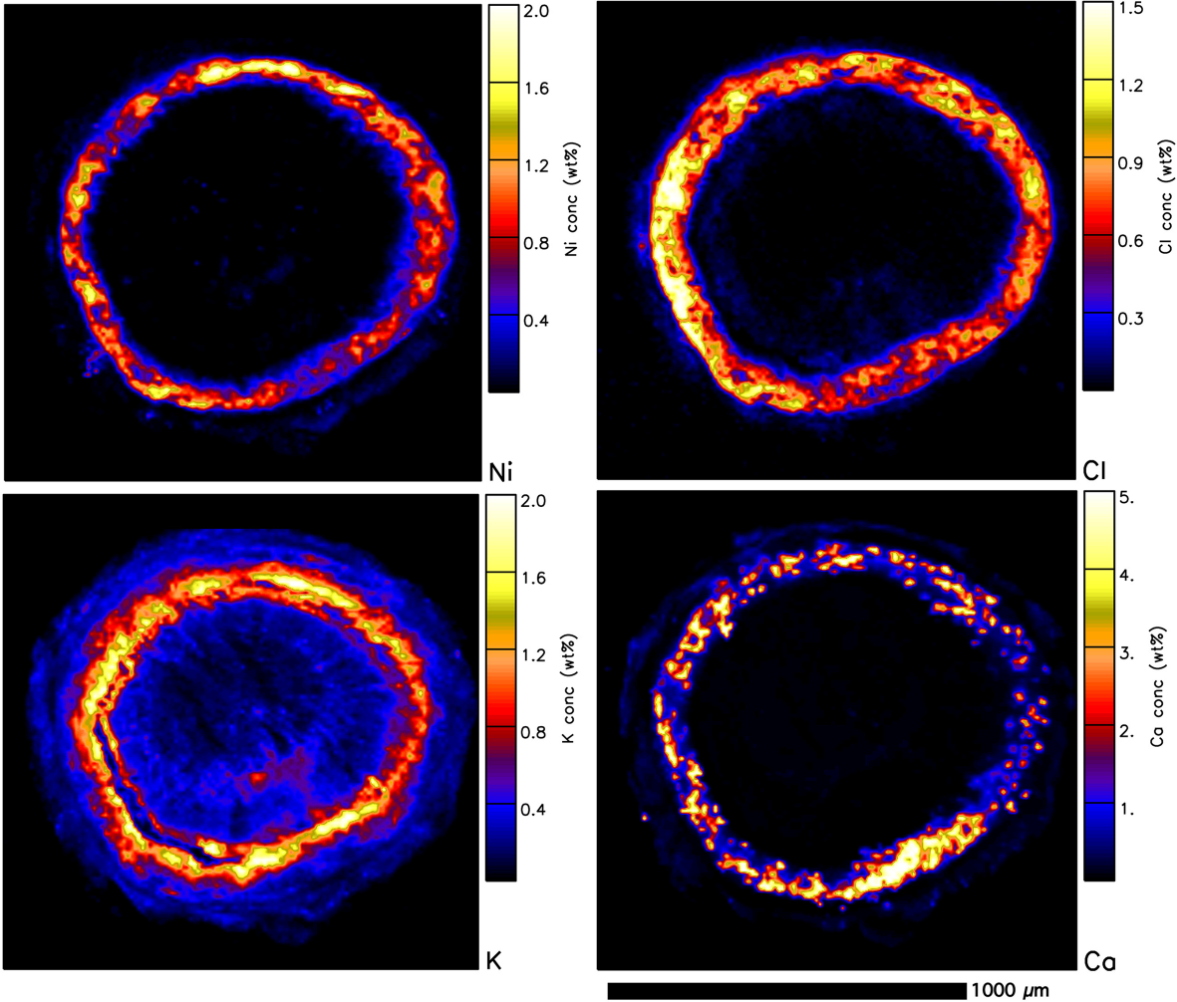
Individual elemental particle-induced X-ray emission (PIXE) maps of a freeze-dried *P. rufuschaneyi* root section showing Ni, Cl, K, and Ca maps. Acquired at the nuclear microprobe facility of iThemba LABS.

**Figure 9.**
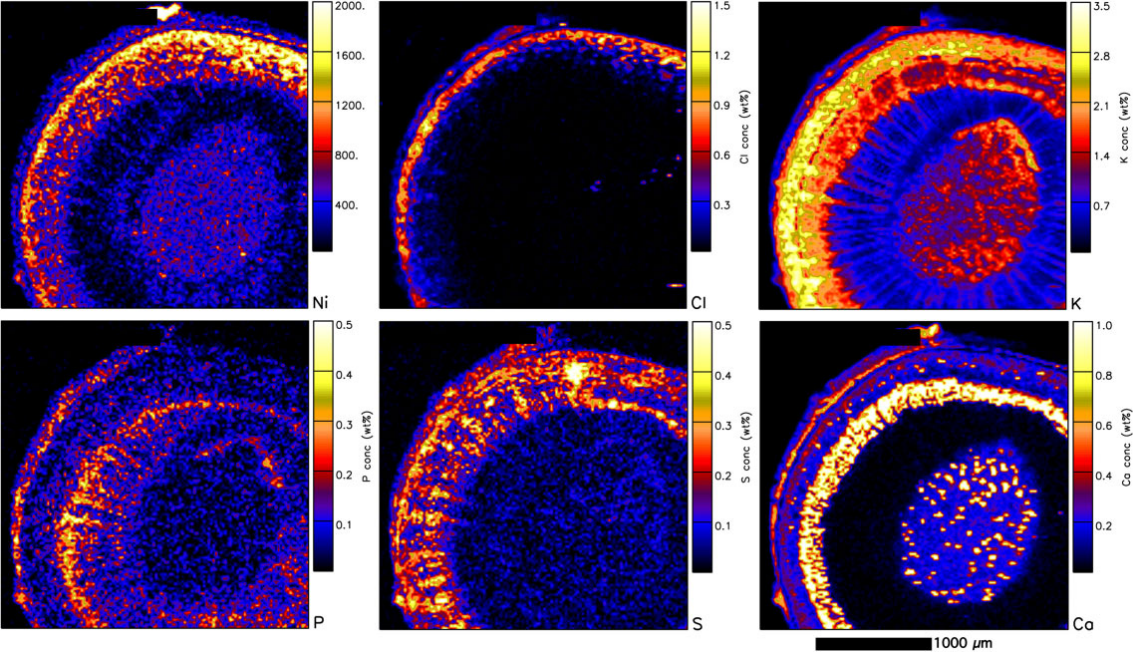
Individual elemental particle-induced X-ray emission (PIXE) maps of a freeze-dried *P. rufuschaneyi* young stem section showing Ni, Cl, K, P, S, and Ca maps. Acquired at the nuclear microprobe facility of iThemba LABS.

**Figure 10.**
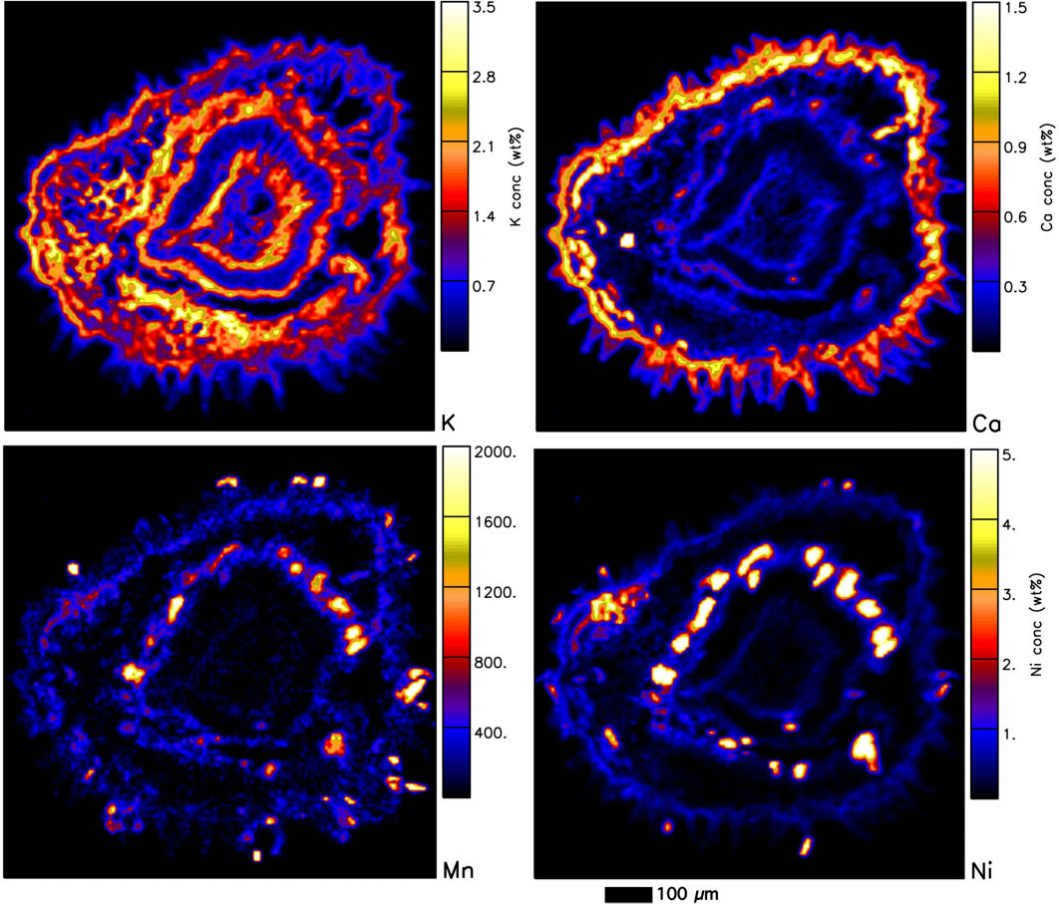
Individual elemental particle-induced X-ray emission (PIXE) maps of a freeze-dried *P. balgooyi* small twig section showing K, Ca, Mn, and Ni maps. Acquired at the nuclear microprobe facility of iThemba LABS.

In addition to the synchrotron XFM analysis on *P. rufuschaneyi* and *P. balgooyi* tissue samples, nuclear microprobe (micro-PIXE) analysis was undertaken on freeze-dried cross-sections of roots, stems, and leaves. Quantitative results are provided in Table 3 (macro elements) and Table 4 (trace elements). In the *P. rufuschaneyi* root (Fig. 8), Ni is concentrated in the phloem and strongly depleted elsewhere (i.e. in the epidermis, cortex, and xylem). Potassium is also concentrated in the phloem as well as in the xylem, much like the distribution of Cl. Calcium also occurs in the cortex, mainly as speckles (likely Ca-oxalate deposits) throughout the cortex. In the young *P. rufuschaneyi* stem (Fig. 9), Ni also occurs in the cortex surrounding the phloem. In young stems of *P. balgooyi* (Fig. 10), Ni is mainly concentrated in the phloem bundles that surround the central pith, whereas the pith itself and the xylem surrounding the phloem bundles are Ni-depleted. The distribution of Co (map not shown) is similar to that of Ni. Manganese is also concentrated in the phloem bundles. Calcium occurs mainly in the periderm and cortex. Potassium is enriched mainly in the xylem and in the cortex. The distribution of Ca marks circular growth rings. Quantitative results of the PIXE analysis are provided in Table 3 (macro elements) and Table 4 (trace elements).

**Table 3.** Nuclear microprobe (PIXE with RBS) quantitative concentration data from samples of roots, twigs, stems, and leaves. Macro-elements (Si, P, S, Cl, K, and Ca). Values in μg g^–1^ dry weight with errors of analysis with ±1 σ uncertainty

	Area	Sample	Si	P	S	Cl	K	Ca
*Phyllanthus*	Small twig	Whole area	<1 330	470 ± 120	1200 ± 110	5960 ± 200	14 390 ± 140	2010 ± 100
*balgooyi*	Small twig	Whole area	<90	580 ± 8	2520 ± 90	10 070 ± 150	15 260 ± 160	3430 ± 75
		Area with high Ni and Co	<1510	<520	975 ± 90	12 580 ± 290	13 970 ± 210	1920 ± 120
	Leaf	Whole area	<100	390 ± 35	1270 ± 50	5660 ± 50	2360 ± 30	14 510 ± 70
		Secondary vascular bundle	<650	640 ± 80	1770 ± 120	11 830 ± 230	3200 ± 60	3690 ± 60
		Secondary vascular bundle	<1190	890 ± 120	1810 ± 210	8060 ± 170	3900 ± 60	4520 ± 80
		Secondary vascular bundle	n.d.	<470	1150 ± 100	7690 ± 270	4790 ± 90	2700 ± 60
		Secondary vascular bundle	<1340	890 ± 140	1380 ± 120	10 700 ± 170	3540 ± 60	3190 ± 70
		Secondary vascular bundle	n.d.	860 ± 260	1300 ± 170	11 160 ± 210	3840 ± 70	2910 ± 60
		Upper epidermis	<580	<200	720 ± 34	4490 ± 90	1130 ± 20	16 190 ± 110
		Lower epidermis	940 ± 180	<240	300 ± 75	560 ± 40	2660 ± 50	34 210 ± 140
		Mesophyll	<310	240 ± 70	1160 ± 70	1630 ± 30	2960 ± 40	28 850 ± 120
	Stem	Whole area	72 ± 51	390 ± 40	1670 ± 65	3140 ± 60	4950 ± 43	1670 ± 20
		Area with high Ni and Co	<390	390 ± 35	1440 ± 60	5300 ± 150	8830 ± 100	2100 ± 40
		Area high in Ni	n.d.	<580	760 ± 140	4840 ± 180	7480 ± 210	3760 ± 100
		Area high in Ni	<1350	<480	810 ± 100	3330 ± 90	7980 ± 100	3060 ± 60
*Phyllanthus*	Old stem	Whole area	1010 ± 170	430 ± 85	360 ± 27	54 ± 7	3790 ± 20	560 ± 12
*rufuschaneyi*		Pith	1210 ± 230	470 ± 95	510 ± 35	37 ± 6	3910 ± 20	940 ± 17
		Secondary vascular bundle	670 ± 180	320 ± 96	390 ± 40	71 ± 13	3610 ± 28	510 ± 13
		Secondary vascular bundle	1060 ± 200	350 ± 60	320 ± 27	65 ± 16	3660 ± 28	644 ± 12
		Secondary vascular bundle	930 ± 190	440 ± 120	420 ± 40	52 ± 16	4750 ± 33	600 ± 11
		Compressed pith	830 ± 210	370 ± 110	770 ± 52	58 ± 26	8810 ± 80	4050 ± 55
	Young stem	Whole area	4610 ± 480	945 ± 90	1190 ± 68	1240 ± 24	15 620 ± 70	4500 ± 50
		Secondary phloem	670 ± 140	1080 ± 100	2430 ± 170	745 ± 40	22 900 ± 160	16 630 ± 150
		Pith	460 ± 100	480 ± 50	570 ± 40	144 ± 10	14 690 ± 70	6000 ± 60
		Xylem	420 ± 70	1590 ± 170	610 ± 30	140 ± 15	9530 ± 40	500 ± 11
		Epidermis	87 630 ± 4 720	1100 ± 190	1490 ± 80	4020 ± 90	12 050 ± 250	5230 ± 110
		Epidermis	79 200 ± 3 600	920 ± 90	1630 ± 60	3340 ± 60	12 120 ± 210	4600 ± 90
		Cortex	440 ± 130	510 ± 60	2660 ± 110	5560 ± 44	28 680 ± 210	3490 ± 60
	Root	Whole area	8700 ± 700	580 ± 54	2760 ± 100	3130 ± 24	5720 ± 110	6700 ± 40

n.d., not determined.

**Table 4.** Nuclear microprobe (PIXE with RBS) quantitative concentration data from samples of roots, twigs, stems, and leaves. Trace-elements (Cr, Mn, Fe, Co, Ni, and Zn). Values in μg g^–1^ dry weight with errors of analysis with ± 1 σ uncertainty

	Area	Sample	Cr	Mn	Fe	Co	Ni	Zn
*Phyllanthus*	Small twig	Whole area	<42	1370 ± 70	<69	290 ± 50	71 620 ± 700	490 ± 60
*balgooyi*	Small twig	Whole area	<1.6	224 ± 7	19 ± 4	27 ± 8	8600 ± 120	113 ± 4
		Area with high Ni and Co	<52	2070 ± 100	<87	630 ± 74	133 400 ± 1600	790 ± 80
	Leaf	Whole area	4.7 ± 0.9	592 ± 14	60 ± 3	18 ± 4	5530 ± 70	33 ± 2
		Secondary vascular bundle	<20	1760 ± 53	142 ± 14	105 ± 15	31 860 ± 310	100 ± 16
		Secondary vascular bundle	<36	1900 ± 70	<54	128 ± 26	41 750 ± 640	<119
		Secondary vascular bundle	<49.	2390 ± 130	135 ± 54	218 ± 41	69 940 ± 930	<175
		Secondary vascular bundle	<43	1500 ± 72	<69	226 ± 36	51 840 ± 530	<147
		Secondary vascular bundle	<57	2860 ± 100	245 ± 71	325 ± 72	60 760 ± 880	<210
		Upper epidermis	<12	283 ± 10	<16	<21	5700 ± 62	<36
		Lower epidermis	<15	92 ± 7	44 ± 10	36 ± 12	10 590 ± 140	<42
		Mesophyll	<6	131 ± 5	32 ± 5	13 ± 4	4110 ± 70	19 ± 3
	Stem	Whole area	<1.7	86 ± 3	15 ± 1	25 ± 4	7660 ± 100	53 ± 2
		Area with high Ni and Co	<12	383 ± 12	30 ± 14	260 ± 25	60 370 ± 630	334 ± 18
		Area high in Ni	<60	195 ± 51	<100	297 ± 55	93 650 ± 1560	400 ± 70
		Area high in Ni	<47	140 ± 24	<83	153 ± 40	61 810 ± 770	<213
*Phyllanthus*	Old stem	Whole area	1.2 ± 0.4	14.4 ± 0.4	13.1 ± 0.6	<0.7	353 ± 7	6.2 ± 0.4
*rufuschaneyi*		Pith	<1.7	22 ± 1	12 ± 1	<1.3	376 ± 7	8.4 ± 1
		Secondary vascular bundle	<5	12	<4	<4	714 ± 25	<7
		Secondary vascular bundle	<6	21 ± 4	26	<5	864 ± 24	9 ± 4
		Secondary vascular bundle	<5	12 ± 3	14	<4	720 ± 18	11 ± 4
		Compressed pith	<9	62 ± 8	<9	<11	2830 ± 80	48 ± 11
	Young stem	Whole area	5.2 ± 0.8	50 ± 2	253 ± 6	<3	620 ± 15	17 ± 1
		Secondary phloem	<3	50 ± 4	6 ± 2	<4	795 ± 17	20 ± 3
		Pith	<2	58 ± 3	18 ± 2	<2	572 ± 11	26 ± 3
		Xylem	<2.0	16 ± 1	8 ± 1	<2	245 ± 9	7 ± 1
		Epidermis	118 ± 15	100 ± 12	3680 ± 150	39 ± 21	460 ± 50	<24
		Epidermis	90 ± 5	131 ± 12	3990 ± 110	23 ± 12	620 ± 30	27 ± 5
		Cortex	<3	65 ± 5	23 ± 4	<8	1420 ± 40	20 ± 3
	Root	Whole area	68 ± 3	70 ± 4	2030 ± 30	12 ± 4	2820 ± 30	41 ± 1

**Figure 11.**
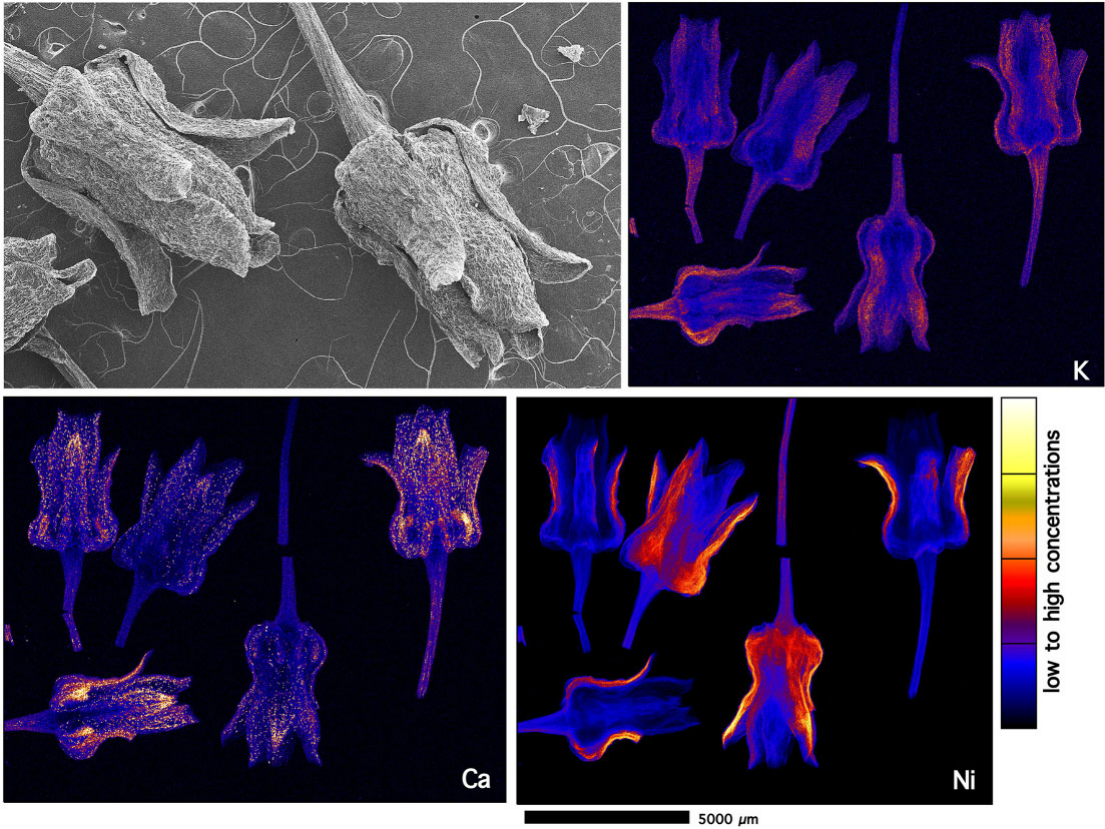
Individual elemental micro-X-ray florescence (μXRF) maps of *P. rufuschaneyi* inflorescences (panels showing K, Ca, and Ni distributions). The elemental image was acquired in a 10-μm step size with a 2.6 ms dwell per pixel. The top-left panel shows a scanning electron microscopy (SEM) image of a dehydrated *P. rufuschaneyi* inflorescence as a visual aid to the μXRF maps. Acquired at the X-ray fluorescence microscopy (XFM) beamline of the Australian Synchrotron (ANSTO).

Nickel in individual inflorescences of *P. rufuschaneyi* (Fig. 11) is mainly located in the base of the petals (evident especially from the angular view in the Ni map of the leftmost flower). *Phyllanthus* flowers are generally monochlamydeous (i.e. do not have a separate calyx and corolla). There does not appear to be a substantial accumulation of Ni in the style or ovary, but enrichment in the receptacle.

## Discussion

This study has added further insights into the ecophysiology of Ni hyperaccumulation in *P. balgooyi* and *P. rufuschaneyi*. The species have in common that their phloem tissue is green from extreme Ni accumulation, and *P. balgooyi* exudates a phloem sap that contains a maximum of 16.9 wt% Ni. In contrast, at the whole organ level, there is Ni enrichment in the leaf lamina in *P. rufuschaneyi* and in the secondary veins of *P. balgooyi.* The strong enrichment of Ni in the vascular bundles of *P. balgooyi* (which is less in *P. rufuschaneyi*) is now known from a number of woody hyperaccumulator plant species from tropical regions, including in members of the Violaceae, such as *Rinorea* cf. *bengalensis* and *R.* cf. *javanica* from Borneo,^40^ and *Hybanthus austrocaledonicus* (Vieill.) Schinz & Guillaumin ex Melchior from New Caledonia, and in the laticifers of *P. acuminata* from New Caledonia,^4^*Euphorbia helenae* subsp. *grandifolia* Borhidi & O. Muñiz from Cuba,^18^ and in *Ficus trachypison* K.Schum. & Lauterb. and *Planchonella roxburghioides* from Indonesia.^60^ Substantial Ni enrichment in the phloem is also found in the South African perennial herbaceous hyperaccumulators *Berkheya zeyheri* Oliv. & Hiern subsp*. rehmannii* (Thell.) Roessler var. *rogersiana* (Thell.) Roessler^61^ and *Senecio coronatus.*^62^

The distinctive enrichment of Ni in the phloem implies substantial redistribution (both downward and upward movements) to other parts of the plants. As such, Ni can be translocated to emerging young shoots. Indeed, experimental work undertaken on *Noccaea caerulescens* (J.Presl & C.Presl) F.K.Mey. using the isotope tracer ^61^Ni revealed that 89% of exported Ni from old leaves moved upward to young leaves, but just 11% moved to the roots.^11^ In the phloem, Ni is complexed primarily with organic acids, specifically with the carboxylate citrate in tropical species.^28^ Nickel is known to be phloem mobile and easily transferred from sources to sinks.^63^ The high enrichment of Ni in the phloem is likely to have a major effect on the osmotic pressure of the sieve elements.^36^

The small (20–50 μm) Ni-rich hotspots found dispersed over the *P. balgooyi* leaf surface, especially towards the tip, are probably deposits emanating from leaf venation terminals in guttation fluids. Guttation is a form of secretion of liquids from the leaves *via* so-called ‘hydathodes’, which are permanently open.^64^ Similar observations of excess Ni excreted from hydathodes have been made in the Ni hyperaccumulators *Odontarrhena chalcidica* (Janka) Španiel, Al-Shehbaz, D.A.German & Marhold (synonym *Alyssum murale*),^65^*Noccaea japonica* (H.Boissieu) F.K.Mey. (synonym *Thlaspi japonicum*)^66^ and in *Glochidion* cf. *sericeum*.^51^

Robinson *et al.*^67^ hypothesized that accumulation of Ni in the upper epidermis could have a function to protect the underlying chlorophyll against harmful ultraviolet radiation. In the epidermal area, accumulated Ni is kept away from physiologically sensitive processes associated with photosynthesis in the palisade mesophyll. Localization in the foliar epidermis could be the result of passive accumulation through the transpiration-driven water stream.^68^ Movement of elements from the soil into plant roots results from convection of the element dissolved in soil solution to the rhizodermis cell membrane where uptake occurs or by diffusion from soil mineral phases of the element to the rhizodermis cell membrane.^69,70^ It is especially intriguing that *Odontarrhena* attains >2 wt% Ni in shoots from a very low concentration of soluble Ni in the soil solution, whereas in nutrient solutions 300 μM Ni is required to attain >1 wt% Ni.^71^ Also, puzzling is the fact that Ni uptake and accumulation in *O. chalcidica* triples when soil pH is changed from 5.5 to 7.5,^72^ a response opposite to ‘normal’ plants. Taken together, this suggests that processes that are yet to be understood at the soil mineral-root endodermis interface are key to the uptake pathways.

## Conclusions

The results show that *P. balgooyi* has extraordinary enrichment of Ni in the (secondary) veins of the leaves, whereas in contrast, in *P. rufuschaneyi*, it occurs in interveinal areas. In the roots and stems, Ni is localized mainly in the cortex and phloem but depleted in the xylem. The findings of this study show that, even within the same genus, the distribution of nickel and other elements, and inferred processes involved with metal hyperaccumulation, can differ substantially between species. The high-resolution and sensitivity (for both hyperaccumulated elements and nutritional elements) of XFM and PIXE have proven to be powerful tools to reveal tissue and cellular-level elemental distribution. This study has added further insights into the ecophysiology of Ni hyperaccumulation in *P. balgooyi* and *P. rufuschaneyi*. Although we now have a comprehensive understanding of the distribution and chemical speciation of Ni at the whole plant level, as well as at the level of tissues and cells, many fundamental questions remain. Uncovering the mechanisms of how hyperaccumulation evolved requires molecular biology investigations, especially in tropical taxa that make up most of the species known globally. Unfortunately, to date there has been very little research effort towards the study of Ni hyperaccumulators, even less on tropical species, and fewer still at the molecular level. Currently, work undertaken on *Psychotria gabriellae* (Baill.) Guillaumin has identified a candidate gene (IREG1, iron-regulated transporter) for Ni tolerance and accumulation.^10^ This was confirmed in a recent study undertaking an RNA-Seq comparison in Ni hyperaccumulator species from New Caledonia and Cuba, which revealed convergent molecular mechanisms with high expression of IREG/Ferroportin transporters linked to Ni hyperaccumulation.^73^ There remains, therefore, much scope for research in this space to identify the molecular pathways of Ni during uptake in the root and the associated cell membrane transporters involved.

## Data Availability

The data underlying this article will be shared on reasonable request to the corresponding author.
